# Multidimensional Regulation of Cardiac Mitochondrial Potassium Channels

**DOI:** 10.3390/cells10061554

**Published:** 2021-06-19

**Authors:** Bogusz Kulawiak, Piotr Bednarczyk, Adam Szewczyk

**Affiliations:** 1 Laboratory of Intracellular Ion Channels, Nencki Institute of Experimental Biology, Polish Academy of Sciences, Pasteura 3, 02-093 Warsaw, Poland; a.szewczyk@nencki.edu.pl; 2 Department of Physics and Biophysics, Institute of Biology, Warsaw University of Life Sciences-SGGW, Nowoursynowska 159, 02-776 Warsaw, Poland; piotr_bednarczyk@sggw.edu.pl

**Keywords:** mitochondria, cardiac tissue, ischemia/reperfusion, mitochondrial potassium channels, cytoprotection, potassium channel openers

## Abstract

Mitochondria play a fundamental role in the energetics of cardiac cells. Moreover, mitochondria are involved in cardiac ischemia/reperfusion injury by opening the mitochondrial permeability transition pore which is the major cause of cell death. The preservation of mitochondrial function is an essential component of the cardioprotective mechanism. The involvement of mitochondrial K^+^ transport in this complex phenomenon seems to be well established. Several mitochondrial K^+^ channels in the inner mitochondrial membrane, such as ATP-sensitive, voltage-regulated, calcium-activated and Na^+^-activated channels, have been discovered. This obliges us to ask the following question: why is the simple potassium ion influx process carried out by several different mitochondrial potassium channels? In this review, we summarize the current knowledge of both the properties of mitochondrial potassium channels in cardiac mitochondria and the current understanding of their multidimensional functional role. We also critically summarize the pharmacological modulation of these proteins within the context of cardiac ischemia/reperfusion injury and cardioprotection.

## 1. Introduction

During ischemia/reperfusion injury, mitochondrial dysfunction is one of the initial triggers of cardiac death. This complex process involves a variety of events, such as changes in reactive oxygen species (ROS) synthesis, calcium ion overload in the mitochondria, disruption of mitochondrial membranes and a lack of ATP synthesis [1,2,3]. It has been known for many years that proper ion homeostasis is the key biophysical process in mitochondria that guarantees that they function properly and thus is key to the proper cell functioning. The potassium cycle, i.e., the process of K^+^ accumulation and efflux from the mitochondria, is an important part of mitochondrial ion homeostasis. It participates in the regulation of ROS synthesis and mitochondrial matrix volume adjustment. The potassium cycle involves an influx of potassium via potassium uniport and potassium efflux via a K^+^/H^+^ antiporter [4,5]. 

**Figure 1:**
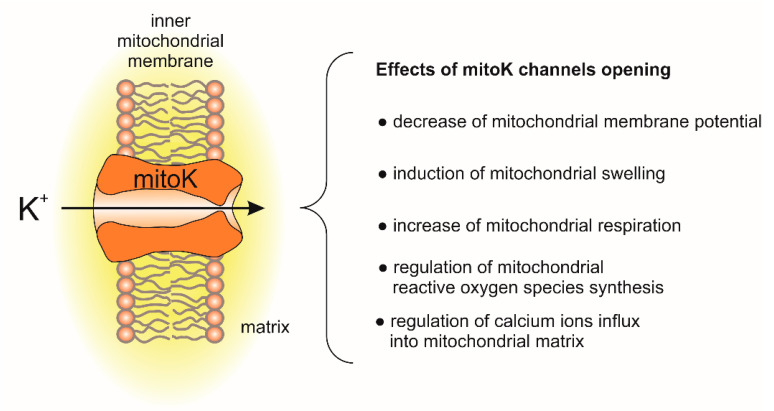
Effects of mitochondrial potassium channel opening on mitochondrial functions.

The molecular identity of potassium uniport was clarified with the identification of potassium channels in the inner mitochondrial membrane [6,7]. Over the last 30 years, few potassium (mitoK) channels have been identified in the inner mitochondrial membrane. The main effects of mitoK channel opening and their impact on mitochondrial functions are presented in Figure 1.

**Figure 2:**
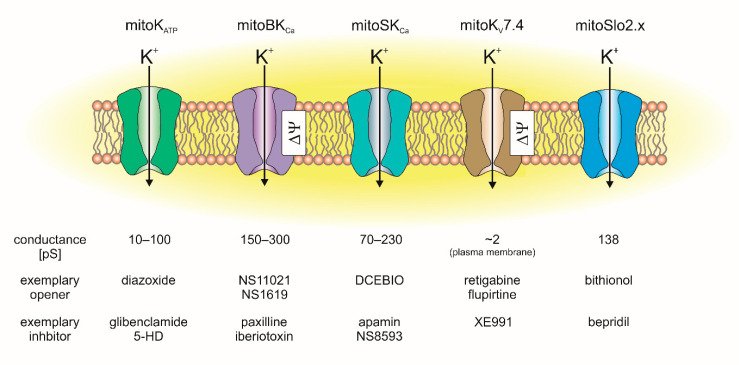
Mitochondrial potassium channels identified in heart tissue: their conductance and exemplary pharmacological tools (channel openers and inhibitors) used in channel characterization.

In cardiac mitochondria, the following mitochondrial potassium channels were identified: the mitochondrial ATP-sensitive potassium (mitoK_ATP_) channel, mitochondrial large-conductance calcium-activated potassium (mitoBK_Ca_) channel, mitochondrial small-conductance calcium-activated potassium (mitoSK_Ca_) channel, mitochondrial sodium-activated potassium (mitoSlo2) channel and mitochondrial voltage-regulated potassium (mitoKv7.4) channel (Figure 2) [7,8,9,10].

Despite many open questions about the functioning of these proteins, the molecular identification of the channels has driven intensive pharmacological characterization studies [9,10,11,12,13]. As mentioned above, heart mitochondria contain several types of potassium channels that are regulated by various factors, including ATP (mitoK_ATP_), calcium ions (mitoSK_Ca_, mitoBK_Ca_), membrane potential (mitoBK_Ca_, mitoKv7.4), pH (mitoK_ATP_) and ROS and redox-dependent pathways (e.g., mitoK_ATP_, mitoBK_Ca_). It is tempting to then ask the question: why do heart mitochondria contain so many potassium influx pathways? Is there any physiological benefit in having channels regulated by so many different ligands? We believe that understanding the spacio-temporal changes in these conditions/ligand concentration will help us to understand the protective role of various mitochondrial potassium channels.

## 2. Overview of Mitochondria in Ischemia/Reperfusion Cardiac Tissue Injury

Reduced blood flow (ischemia) results in a decrease in glucose and oxygen levels, which limits the ability of mitochondria to synthesize ATP. The lowered ATP concentration results in a deregulation of the ionic balance in the cell, which is maintained by ATP-dependent ionic pumps and transporters [1,3,14]. This can result in an increase in calcium ion levels and cellular swelling, membrane disruption and cell death. Mitochondria are the main source of ATP in the cardiac cell and are therefore essential during ischemia/reperfusion injury [1,14,15]. One of the first events that affect the mitochondrion during ischemia is the disturbance in oxidative phosphorylation. Decreasing the concentration of oxygen results in the inhibition of cytochrome *c* oxidase (complex IV) [1,14]. This terminal enzyme of the mitochondrial respiratory chain catalyzes the reduction of oxygen to water [16]. Together with complex I and III, cytochrome *c* oxidase generates an electrochemical gradient across the inner mitochondrial membrane by pumping protons from the mitochondrial matrix to the intramitochondrial space [1,14]. This is possible due to the energy derived from the electrons transported through these complexes, and these electrons come from substrates that are formed during the Krebs cycle or beta oxidation of lipids. The electrochemical proton gradient generated by respiratory chain complexes is utilized by ATP synthase for ATP synthesis. Moreover, a drop in the mitochondrial membrane potential followed by a decrease in ATP synthesis may lead to the reversal of ATP synthase activity and ATP hydrolysis, which restores the mitochondrial membrane potential [1,14,15]. Mitochondrial function is restored during reperfusion; however, several disturbing phenomena may occur that may result in damage to the mitochondria [1,3,15]. One of the key events is the restoration of mitochondrial membrane potential. This can induce an uncontrolled influx of calcium ions, which accumulate during ischemia into the mitochondrial matrix [1,3,14,17,18]. Excessive calcium ions induce both the permeability transition pore (PTP) opening and cell apoptosis [1,19]. On the other hand, succinate accumulated during the ischemic stage can be consumed by respiratory chain complex II, and reverse electron flow from complex II to complex I can be induced [18,20,21]. As a consequence, excessive synthesis of ROS by complex I may occur [2,20,22]. It can also have detrimental consequences such as the induction of permeability transition pore-mediated cell death induction [19,23,24]. This brief description shows that three factors, among others, play a key role in damaging cells during ischemia/reperfusion failure: ATP, calcium ions and reactive oxygen species. Therefore, mitochondria are obvious targets for cardioprotection against cardiac ischemia/reperfusion injury [10,18,23,25]. Finally, during ischemia, anaerobic glycolysis, ATP hydrolysis, and release of protons from acidic organelles cause the pH in cardiac tissues to decrease by one unit or more (≥10-fold increase of hydrogen ion concentration) [26,27,28].

These factors (ATP, Ca^2+^, ROS/redox and pH) regulate the activity of mitochondrial potassium channels. Activating mitochondrial potassium channels can help preserve the functions of the cardiac mitochondria during ischemia/reperfusion. Below, we briefly describe the regulation and molecular composition of the mitochondrial potassium channels found in cardiac mitochondria. We also present examples and briefly explain the possible mechanisms of cardioprotection that are mediated by the activation of cardiac mitoK channels.

## 3. Mitochondrial ATP-Sensitive Potassium Channels

**Figure 3:**
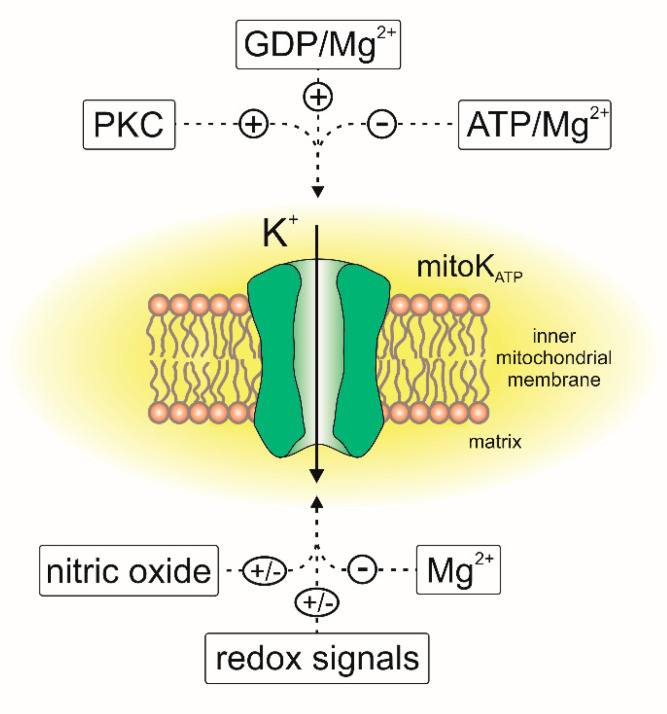
Regulation of the mitoK_ATP_ channels by endogenous factors. “+,” activation of the channel; “–,” inhibition of the channel activity.

Thirty years ago, Inoue et al. described an ATP-sensitive potassium-specific channel present in the inner membrane of rat liver mitochondria [29]. They recorded the flow of potassium through the channel, which was inhibited by 1 mM ATP and 5 µM antidiabetic sulfonylurea—glibenclamide (Figure 3). Later, electrophysiological, pharmacological and biochemical studies showed that ATP-sensitive potassium (mitoK_ATP_) channels were present in the mitochondria of various cell types. These channels have been found in the mitochondria of skeletal muscles [30], brain [31,32], renal tissue [33], human T lymphocytes [34], skin fibroblasts [35] and heart tissue [36,37,38]. The presence of the mitochondrial ATP-sensitive potassium channel has also been reported in nonmammalian mitochondria [39,40,41].

Electrophysiological methods, including the patch-clamp and planar lipid bilayer techniques, have been successfully applied to study the biophysical and pharmacological properties of mitoK_ATP_ channels [29,38,42,43,44]. Most data indicate that these channels have a conductance of approximately 100 pS [34,35,38,45,46]. Recently, it was reported that the mitoK_ATP_ conductance can be close to 60 pS [44]. Additionally, Garlid’s group observed conductance values in the range of 10–30 pS [36,47]. Certainly, the observed differences in conductance between the mitochondrial ATP-sensitive potassium channels could have been a result of various factors, including the tissue used, methodology of measurement and experimental conditions. The observed differences in conductance may also depend on the effective concentration of K^+^; therefore, it should be taken into account when comparing this property of the channel.

With some exceptions, the pharmacology of the mitoK_ATP_ channel is similar to that of the plasma membrane K_ATP_ channels. At low concentrations, diazoxide is believed to be a specific activator of the mitochondrial K_ATP_ channel [48,49,50]. Similarly, BMS191095 opens the mitoK_ATP_ channel at a range of micromolar concentrations [35,51]. It seems that these compounds are highly selective toward the mitoK_ATP_ channel and are considered to be mitochondrial potassium channel openers [7,52]. With respect to mitoK_ATP_ channel inhibitors, ATP is believed to be the major K_ATP_-type channel blocker [29,38,45]. Additionally, the regulation of cardiac mitoK_ATP_ channels by magnesium ions, quinine and nucleotides has been described [6,47,53,54]. Moreover, it can be assumed that the cardiac mitoK_ATP_ channel is regulated by multiple phosphorylation events [45,55]. Cardiac mitoK_ATP_ channels are inhibited by 5-hydroxydecanoic (5-HD) and the antidiabetic sulfonylurea glibenclamide [56]. 5-HD is highly selective toward the mitoK_ATP_ channel and generally does not inhibit the K_ATP_ channel from the plasma membrane [7,45,57]. It is believed that glibenclamide acts through interacting with the sulfonylurea receptor (SUR) subunit to inhibit the mitoK_ATP_ channel [36,44,58]. In contrast, it has been shown that HMR1098 is a potent and selective blocker of the plasma membrane ATP-sensitive potassium channel and does not block mitoK_ATP_ channels [45,58].

Despite many efforts, the molecular structure of the mitoK_ATP_ channel has been a mystery for a long time. Initially, it was thought that the channel (similar to the K_ATP_ channels from plasma membranes) is composed of potassium-specific pore-forming subunits (Kir6.1 or Kir6.2, known as KCNJ8 or KCNJ11) and a regulatory subunit formed by protein-binding sulfonurea derivatives (SUR1 or SUR2 coded by ABCC8 or ABCC9) [7,59,60]. In rat tissue (liver, pancreas, brain, skeletal and heart muscle), the attempts to identify the Kir subunit indicated that it is similar to the Kir6.1 or Kir6.2 subunit [59,61,62,63]. In turn, studies using a fluorescent glibenclamide derivative suggested that the mitoSUR sulfonurea receptor could be a protein with a mass close to 65 kDa [31]. It is worth noting that the mitoSUR receptor differs from its counterparts in the K_ATP_ channels that are found in plasma membranes, as it is a receptor with a low sulfonurea affinity [64]. However, it seems that Kir6.2 is not responsible for mitoK_ATP_ formation. In mouse cardiac tissue with Kir6.2 deletion, mitoK_ATP_ activity is still present [65]. However, the cardioprotective effect induced by diazoxide was decreased. Therefore, it was proposed that this protein plays some role in the cytoprotection induced by diazoxide but it is probably unrelated to the mitoK_ATP_ channel [65].

Later, it was proposed that mitochondrial-targeted isoform 2 of the renal outer medullary potassium channel (ROMK2, also known as KCNJ1 or Kir1.1) protein creates a pore-forming subunit of mitoK_ATP_ in heart mitochondria [66]. Mass spectrometry analysis confirmed the presence of ROMK protein in highly purified cardiac mitochondria. Moreover, expression of ROMK2 protein in cardiac-derived H9c2 cells revealed that the protein reaches mitochondria, and the N-terminal part of the protein was shown to play a role as a mitochondrial targeting sequence [66]. It was also demonstrated that the overexpression of ROMK2 in mitochondria confers protection against oxidative stress-induced cell death. It was expected that identifying the mitoROMK channels will provide molecular targets for therapeutic investigations of the mitoK-induced survival pathway [66]. The channels were inhibited by 5-HD and by tertiapin Q (an inhibitor of ROMK-type channels). Additionally, it has been reported that the mitoK_ATP_ from mitochondria of H9c2 cells that overexpress ROMK2 was inhibited by ATP/Mg^2+^ and activated by diazoxide [38]. Unexpectedly, global knockout of the ROMK potassium channel worsened cardiac ischemia/reperfusion injury but cardiomyocyte-specific knockout did not [67]. In light of these data, the role of ROMK2 in mitoK_ATP_ channel formation remains unclear. The question of the structure of the mitoK_ATP_ channel is all the more interesting as recent data indicate quite convincingly that another protein may contribute to this channel formation.

Namely, it has been proposed that the protein encoded by the CCDC51 gene (NCBI ID 79714) is a pore-forming subunit of mitoK_ATP_ [44]. Using planar lipid bilayers, it has been shown that a complex formed by this protein mediates ATP-sensitive K^+^ currents and has properties typical of mitoK_ATP_. It was also found that the activity of the channel formed by CCDC51 is regulated by the mitoSUR protein, which is responsible for ATP binding. In vitro reconstitution of CCDC51 with mitoSUR recapitulates the main pharmacological properties of mitoK_ATP_; the channel is activated by diazoxide and inhibited by glibenclamide. However, diazoxide sensitivity by the pore is achieved only in the presence of mitoSUR. Overexpression of CCDC51 resulted in a decrease in mitochondrial membrane potential and mitochondrial calcium levels. On the other hand, deletion of CCDC51 in HeLa cells influenced mitochondrial function, including organelle swelling and mitochondrial membrane potential, and it decreased oxidative phosphorylation [44]. Importantly, loss of mitoK_ATP_ suppresses the cardioprotection that is elicited by diazoxide-induced pharmacological preconditioning [44]. Interestingly, it was also suggested that ATP synthase subunits may form a pore of the mitoK_ATP_ channel [68]. Ion channel activity was observed after reconstitution of mammalian heart F_1_F_O_ in KCl-containing proteoliposomes. The channel had properties similar to those of mitoK_ATP_. It was modulated by mitoK_ATP_ activators and inhibitors [68]. Therefore, it was concluded that ATP synthase may serve as a mitochondrial K^+^-uniporter [68].

Many studies have shown that the pharmacological activation of mitoK_ATP_ by potassium channel openers, such as diazoxide, pinacidil and BMS-191095, protects cardiac tissue and reduces the size of infarct after ischemia/reperfusion injury [7,9,52,56,60,69]. Usually, potassium channel openers are applied before the onset of ischemia to induce pharmacological preconditioning [60]. The role of the mitoK_ATP_ channel in protecting the myocardium against hypoxia became undeniable after it was found that diazoxide has higher specificity for the mitoK_ATP_ channel than for the K_ATP_ channel from the plasma membrane of myocardial cells [48]. It was shown that application of diazoxide at concentrations not affecting the plasma membrane K_ATP_ channel activity protects the rat heart from anoxic damage [48]. Administration of 5-HD during quenching with short periods of hypoxia reverses the protective effect, which indicates that the mitoK_ATP_ channel has a role in this process [70]. Using HMR-1098 and 5-HD inhibitors, it was shown that the cardioprotective effect is indeed mediated by mitochondrial channels rather than sarcolemmal K_ATP_ channels [71]. Pharmacological activation of mitoK_ATP_ influences the mitochondrial volume, which prevents the mitochondrial matrix from excessive contracting, and may be an important part of the cytoprotection mechanism [72]. Interestingly, 5-HD inhibited the cardioprotective effect against ischemia/reperfusion injury that was induced by atorvastatin, which is a well-known medicament [73].

Another study showed that the opening of the mitoK_ATP_ channel reduces mitochondrial uptake of Ca^2+^ during ischemia/reperfusion [74]. However, there is evidence that activation of the mitoK_ATP_ channel does not lead to significant mitochondrial depolarization, and it slightly affects Ca^2+^ uptake [75]. Direct evidence of vectorial pH regulation of mitoK_ATP_ channels, with the use of lipid bilayers, was reported [55]. Alkalizing the matrix site increased the cardiac mitoK_ATP_ conductance and the probability of channel opening. On the other hand, acidification of both matrix and intramembrane space compartments resulted in decreased probability of channel opening [55].

The crucial role of mitochondrial ROS was also described as a part of the mitoK_ATP_-related cardioprotection [53,56]. Several studies have shown that during short periods of hypoxia, there is an increase in ROS synthesis, and they also showed that 5-HD inhibits this phenomenon [76]. It has also been shown that ROS signaling is necessary for the cardioprotective effects of diazoxide [77]. It was also shown that activation of mitoK_ATP_ stimulates the ROS synthesis by complex I in cardiac mitochondria [78]. The activity of the channel is also regulated by ROS-dependent pathways. The increased level of ROS leads to the activation of protein kinase C (PKC), and PKC promotes mitoK_ATP_ channel activation [79,80,81]. The channel is regulated by various redox pathways and its activity is regulated directly by reactive oxygen and nitrogen species [53,82,83]. It has been reported that oxidative stress results in the activation of the mitoK_ATP_ channel and that this activation can be inhibited by 5-HD or the sulfhydryl alkylating compound N-ethylmaleimide [42]. On the other hand, inhibiting tyrosine kinases and ROS synthesis reverses the protective effect of diazoxide, suggesting that the activation of mitoK_ATP_ is a part of the ROS-mediated signaling pathway [70,81]. There is also evidence that activating the mitoK_ATP_ channel in isolated mitochondria reduces ROS synthesis [53,84]. Modulation of the channel by nitric oxide was also described. It was observed that NO may activate the cardiac mitoK_ATP_ channel directly or indirectly, and this is important for cardioprotection [85,86]. However, direct patch-clamp experiments have shown that NO reduces the probability of mitoK_ATP_ opening in the Jurkat cells (Figure 3) [34]. It was also found that the mitoK_ATP_ channel might be structurally and functionally coupled with complex II of the respiratory chain [87,88]**,** which opens possibilities for alternative channel regulation pathways. A similar functional linkage was found for the other mitoK channels as described below.

The above examples clearly show that the connection between various redox reactions and mitoK_ATP_ channels in the phenomenon of ischemic preconditioning is complex [7,53]. The observed differences most likely result from the complexity of the ischemia/reperfusion phenomenon as well as the dynamics and magnitude of ROS synthesis during various stages of this process. As outlined below, this conclusion applies not only to the mitoK_ATP_ channel but also to the other mitochondrial potassium channels.

It is important to point out that most of the mitoK_ATP_ modulators mentioned above have side effects and alternative targets in the cell. This should be considered when interpreting the experimental results [6,89]. The opening of the mitoK_ATP_ by diazoxide upregulates the expression of STIM1 and Orai1 by de novo synthesis with a mechanism that involves NF-κB, c-Fos and ROS via MAPK/ERK signaling [90]. It was also shown that the mitoK_ATP_ opener BMS190195 can protect cells via mitoK_ATP_-independent mechanisms [91]. However, it was also shown that BMS191095 has neuronal toxicity [51,56].

## 4. Mitochondrial Calcium-Activated Potassium Channels

### 4.1. Large-Conductance Calcium-Activated Potassium Channels

As mentioned above, calcium ion homeostasis is disrupted during ischemia/reperfusion events. Cardiac tissue mitochondria contain two potassium channels that can be treated as calcium sensors since they are activated by calcium ions. The first channel belonging to this group was the large-conductance calcium-activated potassium (mitoBK_Ca_) channel, which was found in mitochondria of mammalian heart tissue and in cardiac-derived cell lines [11,38,92,93,94,95]. These channels are also present in the mitochondria of other tissues such as brain, skeletal muscle, bronchial epithelium or skin fibroblasts [96,97,98,99,100,101,102,103]. BK_Ca_ channels are also present in other organelles [101,102,104]. However, it is believed that in cardiomyocytes, BK_Ca_ channels are present exclusively in mitochondria and are not found in the plasma membrane [11,105]. Here, we briefly present key findings that describe cardiac mitoBK_Ca_ channels, and more details are presented in excellent papers [11,105,106].

The properties of natively expressed mitoBK_Ca_ channels have been successfully studied through the patch clamping of mitoplasts, and after reconstitution in lipid bilayers isolated from various tissues and cell types [92,93,99,107,108,109,110,111,112].

The basic pharmacological and biophysical properties of the channel correspond to the properties of the BK_Ca_ channels from the plasma membrane. Electrophysiological studies have shown that the cardiac mitoBK_Ca_ channel conductance is between ~150 and ~300 pS [38,92,110,113,114]. However, the first study describing mitoBK_Ca_, which was in the heart mitochondria of guinea pigs, showed four channel conductance levels ranging from ~25 to ~300 pS. The authors reported that within the same recording period, transitions between the conductance levels were observed [92]. The channel activity was voltage dependent, which means that the open probability of the channel depends on the applied voltage. In this case, the channel open probability was low at negative voltages and increased gradually when a more positive voltage was applied [38,92,110]. However, it should be mentioned that the channel recordings are usually performed in the quite narrow range of voltages (usually between −60 and +80 mV) [38,92,110]. In our hands, application of higher voltage usually results in the instability of the mitoplast membrane. By contrast, recordings of the plasma membrane BK_Ca_ are usually performed in a broader range of applied voltages [115,116]; therefore, some differences between the plasma and mitochondrial recordings may result from an insufficient spread of the applied voltages. Additionally, the recording conditions of mitoK channels do not fully correspond to the physiological situation since the membrane potential of fully coupled and functional mitochondria is typically in the range of approximately −150 to −200 mV and is negative on the matrix side.

**Figure 4:**
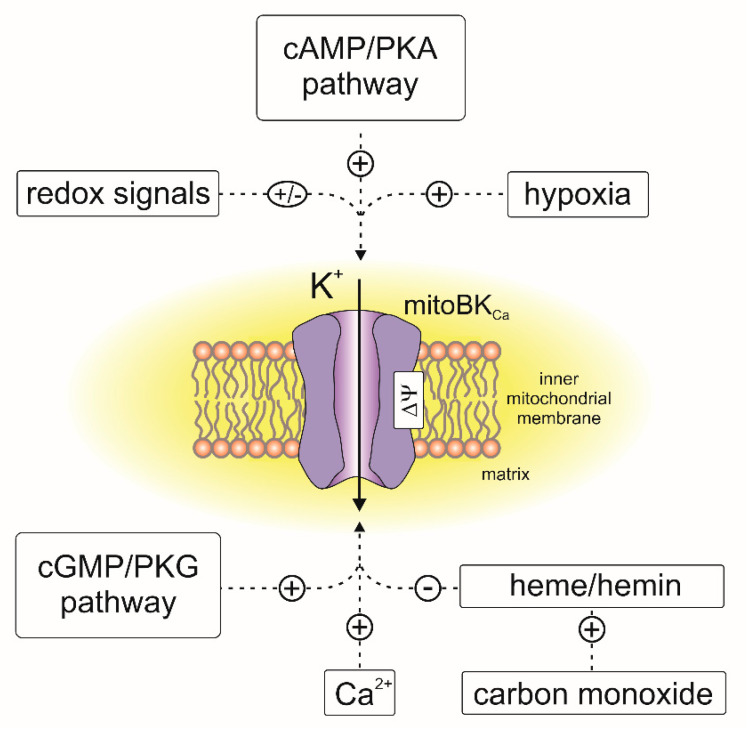
Regulation of the mitoBK_Ca_ channels by endogenous factors. “+,” activation of the channel; “–,” inhibition of the channel activity.

Electrophysiological experiments revealed that mitoBK_Ca_ channels from various tissues are activated by well-known BK_Ca_ channel openers [117,118], including NS1619, NS11021, CGS7184, 12,14-dichlorodehydroabietic acid (diCl-DHAA) and 17β-estradiol [93,97,110,114,117,119,120,121]. Recently, natural flavonoids such as naringenin were described as activators of mitoBK_Ca_ from cardiac tissue [122]. The activity of the channel is inhibited by the peptides iberiotoxin [99,107], charybdotoxin [92,97,109] and diterpene paxilline [38,110,114,121]. MitoBK_Ca_ channels, similar to BK_Ca_ from the plasma membrane, can be regulated by other endogenous molecules, such as heme, hemin and gasotransmitters such as CO, H_2_S and NO [86,117,123,124,125]. It should be mentioned that activation of mitoBK_Ca_ by CO was observed only in the presence of heme [123]. An overview of the channel regulation by endogenous factors is presented in Figure 4.

The activity of the mitoBK_Ca_ channel is modulated by calcium ions, which is typical for BK_Ca_ channels from the plasma membrane [126]. The highest channel activity is observed in the presence of Ca^2+^. Activation of the channel is observed already in the low micromolar concentration range of free calcium. A decrease in calcium ion concentration results in a decrease in mitoBK_Ca_ activity [92,105,110]. This effect is reversible, which means that an increase in calcium ion concentration results in a higher probability of the channel opening. Mitoplast patch-clamp data suggest that C-terminal part of the channel, which contains the calcium-sensing domain, is located in the mitochondrial matrix [92]. This α subunit topology was confirmed by applying the channel inhibitor charybdotoxin from the intramembrane site, which resulted in channel inhibition [92]. On the other hand, calcium ions activated the channels when it was applied from the matrix site [92,110]. Similar observations were reported in mitochondria from other tissues and cell types [98,107,109,120]. These observations suggest that activation of the channel occurs after the influx of calcium ions into the mitochondrial matrix. This mitoBK_Ca_ property may be important for mitochondrial Ca^2+^ handling and it was proposed to be one of the key elements of the cardioprotection mechanism.

Activation of the channels by pharmacological openers can also be manifested by changes in the mitochondrial functions. Activation of the channel results in an influx of K^+^ into the mitochondrial matrix, causing a decrease in mitochondrial membrane potential followed by an increase in oxygen consumption [127]. It was also observed that an increase in mitochondrial flavoproteins autofluorescence occurred as a result of the increase in respiratory chain activity after mitoBK_Ca_ activation [93,121].

Based on the biophysical properties of the mitoBK_Ca_ channel, it has been concluded that the pore-forming subunit of the channel should be encoded by the same gene as the BK_Ca_ in plasma membrane. BK_Ca_ channels are composed of pore-forming α subunits encoded by the KCNMA1 gene (Slo1). The channel is formed by four α subunits [128]. The activity of the pore can be regulated by auxiliary β, γ and Lingo1 regulatory subunits [128,129,130]. Four β1–β4 regulatory subunits have been described that are encoded by the KCNMB1, KCNMB2, KCNMB3 and KCNMB4 genes, respectively [128]. Antibodies recognizing plasma membrane subunits were used to analyze the mitochondrial fractions, which confirmed the presence of the BK_Ca_ α subunit in the inner mitochondrial membrane of mammalian cardiac tissue [92,95,113,131,132] and a heart-derived cell line [132,133]. Quantitative analysis of KCNMA1 gene expression, which encodes the α subunit of the channel, revealed that the gene was expressed in human, mouse and rat cardiomyocytes, but the expression level was significantly weaker in comparison with that in brain tissue or aorta [93]. A large number of BK_Ca_ pore-forming subunit isoforms have been described; therefore, it was unclear which isoform is present in mitochondria [134]. Several studies have suggested that the VEDEC BK_Ca_ isoform locates in the inner mitochondrial membrane [135,136]. The specificity of this isoform is defined by the C-terminus modification. In cardiac cells, this isoform exclusively targets mitochondria; however, in other cell types, it can also reach other cellular compartments [136,137,138,139].

It has also been found that the β1 subunit colocalizes with the cardiac mitochondria of mammalian cells [93,110,132]. In the heart-derived H9c2 cell line, both β1 [132] and β4 proteins were found in the mitochondrial fraction [133]. It has been shown that in mouse cardiomyocytes, the pore-forming subunit interacts with the β1 subunit [110]. In the β1 subunit knockout cells, activation of the channel by calcium ions occurred only when a high positive voltage was applied. By contrast, the channel of mitoplasts isolated from wild-type heart tissue was activated by calcium ions even when a negative voltage was applied [110]. In the same study, two channel populations were found to have different biophysical properties. A possible cause may be the presence of regulatory subunits [110]. It was also found that co-expression with β1 resulted in a higher density of BK_Ca_ in mitochondria [110]. There are still questions on the detailed mechanism of how mitoBK_Ca_ subunits target the mitochondrial inner membrane.

It was found that the β1 subunit might interact with cytochrome *c* subunits in rat cardiac mitochondria [93]. Another study suggested that β1 interacts with complex I and complex V subunits [140]. Mass spectrometry analysis of the cardiac mitoBK_Ca_ channel revealed interactions with mitochondrial translocases, including the TOM complex and carriers and transporters. These included adenine nucleotide translocator, proteins involved in fatty acid metabolism and proteins involved in oxidative phosphorylation, including Krebs cycle proteins and respiratory chain components [11,141]. It was suggested that mitoBK_Ca_ channels had similar interactions in brain mitochondria [138]. In a study describing the global interactions of mouse cochlea BK_Ca_ channels, approximately 20% of the potential partners were related to mitochondrial membranes and the matrix [135]. In glioma cells, respiratory chain activity was found to regulate the mitoBK_Ca_ channels. Increased activity of the respiratory chain decreased the probability of channel opening [107]. However, it is unclear whether a similar regulation exists in cardiac mitochondria.

Similar to mitoK_ATP_ channels, mitoBK_Ca_ channels are also targets for pharmacological intervention. The cardioprotective effect of the mitoBK_Ca_ opening was observed in a model of ischemia/reperfusion heart injury of mammalian tissue derived from various species including guinea pigs, rats, mice and rabbits [92,95,131,142]. Pretreatment with the mitoBK_Ca_ channel opener NS1619 reduced the infarct size of guinea pig hearts and this effect was reversed by paxilline [92]. Similarly, in a study involving the chronic hypoxia of rat cardiomyocytes, NS1619-mediated mitoBK_Ca_ activation increased cell survival [142]. Another study showed that NS1619 prevented ouabain-induced mitochondrial depolarization and cell death in guinea pig ventricular myocytes, and these effects were reversed by pretreating it with paxilline [94]. It was also noted that NS1619 induced mitochondrial flavoprotein oxidation in a paxilline-dependent manner, which suggested that there was mitoBK_Ca_ activity [94]. Cardioprotective effects mediated by mitoBK_Ca_ activation were also observed after NS11021 was applied in isolated perfused rat hearts [119] and isolated rat cardiomyocytes [143]. Cytoprotection of rat cardiomyocytes was also observed after activation of the mitoBK_Ca_ channel by 17β-estradiol [93]. In addition to the canonical activators of the BK_Ca_ channels, the application of flavonoids can also induce mitoBK_Ca_-mediated cardioprotection. Naringenin protected Langendorff-perfused rat hearts functioned against ischemia/reperfusion injury in a paxilline-dependent manner [122,132]. In the H9c2 cell line, the application of naringenin protected the senescent cells against hypoxia-induced cell death, and these effects were reversed after mitoBK_Ca_ inhibition [132]. In the same cell line, it was found that mitoBK_Ca_ channels were involved in the adenosine A1 receptor-induced pharmacological preconditioning against hypoxia-induced cell death [133]. The effect of mitoBK_Ca_ activators on the function of the mitochondrion may change with age. Increasing the age reduces the opening effect of mitoBK_Ca_ on mitochondrial respiration in rat cardiac tissue [144]. The effects of ischemic preconditioning or anesthetic preconditioning seem to be age dependent [145,146]; therefore, it was suggested that the cytoprotective activation potential of mitoBK_Ca_ against an ischemia/reperfusion injury may decrease with age [144,147]. Interestingly, inducing H9c2 cell senescence resulted in a decrease in both α and β1 levels in mitochondrial membranes, which may support the previous idea [132].

The global- and cardiomyocyte-specific knockout of BK_Ca_ channels in mice resulted in a loss of the ischemic preconditioning of heart tissue [113,114]. On the other hand, genetic activation of the channel in the deletion strain preserved the recovery of cardiac function during reperfusion [139]. Mild changes were found in cardiac mitochondrial function after deletion of the KCNMA1 gene by changes in respiration or ROS synthesis [113]. In neuronal tissue, expression of the loss-of-function BK_Ca_ mutants also disturbed mitochondrial function [148].

Several signaling pathways upstream of channel activation were described as triggers of the cardioprotection mechanism mediated by mitoBK_Ca_. For example, it was found that the channel may be targeted by protein kinase A (PKA) [53,94,131]. PKA activators significantly increased NS1619-induced effects on mitochondrial function, and this was not observed in the case of protein kinase C [94]. Furthermore, cilostazol, a PKA activator, induced cardioprotection via the direct activation of mitoBK_Ca_ channels [149]. Recent studies have shown that mitoBK_Ca_ may be a target for a nitric oxide-sensitive guanylyl cyclase/cGMP-dependent protein kinase type I signaling pathway. Direct activation of the channel after phosphorylation by protein kinase G (PKG) was observed in mouse cardiac mitochondria [114]. The involvement of PKG signaling in mitoBK_Ca_-induced cardioprotection was supported by the observations that the application of sildenafil and tadalafil had reduced the infarct size after ischemia/reperfusion had occurred in rat and mouse hearts [150]. Deletion of cardiac BK_Ca_ resulted in loss of the protective effects of both drugs [114]. Moreover, inhibition of nitric oxide synthase decreased cardioprotection in the wild-type but not in hearts with KCNMA1 deletion [114]. Deletion of nitric oxide-sensitive guanylyl cyclase reduced the sildenafil cardioprotective effect; however, pharmacological activation of the channel by NS11021 still reduced ischemia/reperfusion injury [151].

Reactive oxygen species play a key role in cardioprotection induced by mitoBK_Ca_ opening. Pharmacological activation of mitoBK_Ca_ resulted in guinea pig heart preconditioning, which translated into the preservation of heart function and a reduction in infarct size in a model of induced ischemia/reperfusion injury [152]. Reduced mitochondrial Ca^2+^ and normalized NADH levels were also observed in these experiments. Decreased mitochondrial calcium ions levels after mitoBK_Ca_ opening were also observed in rat ventricular myocytes [121,153]. Additionally, lowered superoxide levels were observed in NS1619-treated heart tissue during ischemia and throughout the reperfusion phase [152]. Dismutation of the superoxide to H_2_O_2_ decreased NS1619-induced cardioprotection, suggesting an important role of this radical in mitoBK_Ca_-mediated preconditioning [152]. Additionally, hydrogen peroxide induced the downregulation of β1 [140]. It was also observed that activation of cardiac mitoBK_Ca_ could reduce mitochondrial ROS synthesis during the reverse electron transfer process [127,139]. A similar effect was observed in brain mitochondria, which suggests that this mechanism is universal [154]. Elevated reverse electron flow-driven ROS synthesis was demonstrated to occur during the reperfusion phase as a result of the accumulation of succinate in mitochondria during ischemia [21]. Therefore, the decrease in ROS synthesis as a result of mitoK channel opening seems to be one of the important elements of the cardioprotective mechanism. Preincubation of mitochondria in anoxic condition followed by reoxygenation resulted in a more significant increase in ROS synthesis by mitochondria with deletion of BK_Ca_ than mitochondria from wild-type tissue [113]. On the other hand, it was found that a transient increase in ROS during mitoBK_Ca_ preconditioning may be important in the induction of pro-survival signaling in cardiac tissue. In a model simulating ischemia/reperfusion injury of isolated ventricular myocytes, a slight increase in ROS was detected after mitoBK_Ca_ was activated with NS11021, and this was followed by an increase in cell survival. The application of antioxidants prevented mitoBK_Ca_-mediated cardioprotection [143]. In a human liver cancer cell line, activation of the channel-induced mitochondrial release of ROS was observed, which supports the idea that ROS signaling activation occurs after the opening of the mitochondrial potassium channel [155].

The activity of the mitoBK_Ca_ channel was found to be regulated by hypoxia. Patch-clamp studies revealed that in glioma cells, mitoBK_Ca_ was activated by hypoxia; however, hypoxia had the opposite effect on BK_Ca_ from the plasma membrane [156,157,158]. In rat cultured cardiomyocytes, it was found that hypoxia-induced downregulation of the β1 subunit. A shift from normoxic to hypoxic conditions resulted in a decrease in KCNMB1 gene expression, which was also reflected at the protein level [140]. Silencing of the Hif-2 factor prevented the hypoxia-induced downregulation of β1 [140]. Downregulation of β1 increased the cardiac cell resistance against the cytotoxic effects of ischemia [140]. Another study revealed that chronic hypoxia of rat ventricular myocytes did not change the level of β1 expression, but deglycosylation of this subunit was observed [142].

Despite a large number of studies describing that cardiomyocyte mitoBK_Ca_ participate in cardioprotection, there are data that question the role of the channels in this phenomenon [159]. It was suggested that in isolated hearts of mice, the cardioprotective effect may be mediated by the BK_Ca_ channels of cardiac neurons and not the cardiomyocytes [160]. In a model of isolated cardiomyocyte ischemia/reperfusion injury, NS1619 and NS11021 induced cytoprotection in cells with a KCNMA1 deletion [160]. On the other hand, alternative targets for BK_Ca_ openers in mitochondria and other organelles have been described [118,161,162,163]. It was also suggested that cytoprotection induced by BK_Ca_ modulators may be unrelated to channel opening [164]. Unspecific effects were also observed of BK_Ca_ channel inhibitors such as paxilline. For example, paxilline induced swelling and respiration in an unspecific manner in heart and liver mitochondria [165]. Additionally, it was shown that paxilline induced neuroprotection without the involvement of BK_Ca_ channels [166]. Additionally, some mitoBK_Ca_ activators were shown to induce cytotoxicity [120,167].

### 4.2. Small-Conductance Calcium-Activated Potassium Channels

Another group of calcium-regulated channels found in the mitochondria of cardiac tissue are the small-conductance potassium (mitoSK_Ca_) channels [168]. The small-conductance calcium-activated potassium channel (SK_Ca_) family consists of three members—SK1, SK2 and SK3, and they are expressed in various tissues, including the smooth muscle, brain and heart. The channels are encoded by three KCNN1-3 genes. The common and unique feature of all three SK channels is that they are inhibited by the bee venom toxin apamin. In heart tissue, all three SK1-SK3 isoforms are expressed. The activity of the channel is regulated by phosphatidylinositol bisphosphate [169]. The calcium sensitivity of these channels is likely due to the calmodulin associated with the C-terminal part of the channel [169,170]. For a detailed description of the regulation and function of these channels, see the review by Zhang et al. [170].

In addition to being located in heart mitochondria, mitoSK_Ca_ channels were also identified in the mitochondria of neuronal cells [171,172,173]. Mass spectrometry analysis of calmodulin-bound proteins showed the presence of SK_Ca_ channel peptides in the mitochondria of guinea pig heart. Western blot analysis and confocal microscopy experiments confirmed the mass spectrometry data using anti-SK_Ca_ antibodies that recognized SK3 channels. Further analysis using electron microscopy and immunogold labeling with an anti-SK2 antibody also showed that the SK_Ca_ channels were localized in the inner mitochondrial membrane [168]. However, due to the high homology of various SK_Ca_ isoforms, antibody-based experiments were further supported by detailed analysis of the expression of SK_Ca_ isoforms in heart tissue. It was found that guinea pig cardiomyocytes expressed both SK2 and SK3 channels, whereas in human ventricular tissue, just the expression of SK3 was found [174]. Further analysis confirmed that guinea pig mitochondria contained the SK3.1 isoform, and in human mitochondria, there was an N-terminally truncated SK3.2 splice variant [174]. Western blot analysis confirmed the existence of a truncated isoform of SK3 in purified human cardiac mitochondria [174]. Additionally, mitochondrial localization of the SK3 protein in human and rat cardiomyocytes was also confirmed with electron and confocal microscopy [174]. Expression of the full-length and truncated guinea pig SK3.1 protein in HL-1 suggested that the N-terminal part of the protein was not required for mitochondrial targeting. It was hypothesized that the C-terminus (probably the calmodulin-binding domain) might be important for mitochondrial localization of mitoSK_Ca_ channels [174].

The above findings were supported by electrophysiological recordings of channel activity. The purified protein was incorporated into the planar lipid bilayer, which allowed the activity of individual channels to be observed. The observed activity was inhibited by apamin. The probability of the channel being open was also dependent on the Ca^2+^ concentration. These observations showed that mitoSK_Ca_ channels share basic properties with plasma membrane SK_Ca_ channels. However, it was suggested that apamin and calcium binding sites were localized to the same side of the channel. However, apamine binds to the plasma membrane SK_Ca_ channel on the opposite side of the calcium ions [169]. This may suggest some structural differences between mitoSK_Ca_ and its plasma membrane counterpart. Moreover, multiple conducting states of the channels were reported: 70 pS, 180 pS, 230 pS and 730 pS. These values were higher than the typical conductance of the SK_Ca_ channels, which were close to 2–25 pS [147]. The conductance of the channel was also dependent on the concentration of calcium ions [168].

Functional studies using isolated mitochondria showed an increase in potassium influx into the mitochondrial matrix after the application of channel openers, and the influx was blocked by a mitoSK_Ca_ channel inhibitor [168]. It was also observed that calcium ions that were added externally had stimulated an influx of K^+^ into the mitochondrial matrix. This effect was also inhibited by the SK_Ca_ channel blocker UCL1684 [174]. Interestingly, a decrease of mitoSK_Ca_-mediated K^+^ influx into the mitochondrial matrix was observed when the calcium uptake into the mitochondrial matrix was prevented by inhibition of the mitochondrial calcium uniporter. These experiments suggested that the calcium-sensing domain of the channel is located in the mitochondrial matrix. However, a previous study using mitoplast patch-clamp experiments suggested that the calcium-sensing domain of the mitoSK2_Ca_ channel in HT-22 neuronal cells is located in the intramembrane space [171]. This conclusion was based on a lack of channel inhibition by apamin when it was added to the external medium. These contrary observations suggest the need for a deeper analysis of mitoSK_Ca_ channel topology in mitochondrial membranes.

Experiments performed with an isolated heart model of guinea pigs revealed that application of the channel opener DCEBIO reduced the injury induced by ischemia/reperfusion [168,174,175]. The same was observed in in vivo rat heart studies [174]. It was observed that preconditioning with the opener resulted in improved systolic–diastolic left ventricular pressure and coronary flow, and it decreased the diastolic LV pressure. Additionally, improved cardiac efficiency and a marked decrease in infarct size were reported. These effects were reversed by the SK_Ca_ channel inhibitor NS8593. Additionally, it was noted that the beneficial effects of DCEBIO were abrogated by TBAP, which is a synthetic superoxide dismutase mimetic. This suggested that the reactive oxygen species have a crucial role in SK_Ca_-induced cardioprotection. Importantly, direct functional analysis of mitochondria isolated from guinea pig hearts that were preconditioned with the SK_Ca_ channel opener revealed that the oxygen consumption rate was maintained after ischemia/reperfusion [174,175]. Overexpression of SK3 resulted in the increased survival of the HL-1 cells that were exposed to hypoxia/reoxygenation, and the C-terminal part of the protein beyond the calmodulin-binding domain is crucial for this effect. On the other hand, silencing the SK3 channels in HL-1 cells and the rat H9c2 cardiac myoblast cell line resulted in damage induced by hypoxia/reoxygenation being enhanced [174].

The activation of cytoprotective mechanisms by mitoSK_Ca_ opening has also been described in neuronal and cancer cells [12,172,176,177]. Pharmacological intervention involving SK_Ca_ and mitoSK_Ca_ channels is also discussed as a potential therapeutic strategy in neurodegenerative disorders [147].

## 5. Sodium-Activated Potassium Channels (mitoSlo2)

MitoBK_Ca_ (Slo1) channels are not the only group of large-conductance potassium channels in cardiac mitochondria. Sodium-activated potassium (K_Na_) channels that belong to the Slo2 family are present in the inner mitochondrial membrane [178,179]. These channels are widely expressed in the nervous system, but they have also been described in cardiac tissue, including guinea pig, rat and mouse cardiac tissue [178,180,181].

In mammalian cells, two channels of this family were described: the Slo2.1 (alternatively named Slick, encoded by the KCNT2 gene) and Slo2.2 (also known as Slack, encoded by the KCNT1 gene). Both channels share some similarities with the Slo1 channels. The unitary conductance of both channels is intermediate; for Slack channels it is close to 180 pS and for Slick it is approximately 140 pS [178]. Interestingly, it was found that Slo2 and Slo1 channels can interact and form intermediate channels that are sensitive to calcium ions [182].

Sodium-activated potassium channels were found in the mitochondria of the mouse heart. Bithionol was found to activate thallium cations uptake in isolated mouse heart mitochondria in the presence of ATP. This effect was blocked by paxilline but was not dependent on the presence of calcium ions [183]. Moreover, the application of iberiotoxin, charybdotoxin and apamin (SK_Ca_ channel blocker) did not inhibit uptake. Surprisingly, thallium fluxes were inhibited by bepridil, which targets several channels, including voltage-activated Ca^2+^ channels, K_ATP_ channels from the mitochondria and sarcoplasmic reticulum and sodium-activated potassium channels [180,184,185]. These observations suggested the presence of a new class of channels in cardiac mitochondria that belong to the Slo2 family. This assumption was confirmed when bithionol-stimulated Tl^+^ uptake in Slo1 gene knockout mitochondria in the presence of ATP [183]. It was also found that the anesthetic preconditioning of perfused mouse hearts by isoflurane was not sensitive to loss of the Slo1 gene product. However, paxilline still inhibited anesthetic preconditioning (APC)-induced cardioprotection. Similarly, Slo1 knockout did not influence ischemic preconditioning and the bithionol-stimulated, paxilline-sensitive cardioprotection of the mouse heart [183]. Another support for mitochondrial Slo2 came from experiments performed with *C. elegans*, in which the expression of K_Na_ channels was previously described [186]. Deletion of the Slo2 gene in worms resulted in loss of the APC that was induced by isoflurane. Further experiments showed a decrease in bithionol-induced thallium cations uptake in mitochondria that was derived from the Slo2 gene knockout strain. It was also shown that bithionol-induced Tl^+^ uptake was inhibited by paxilline and bepridil. This effect was not blocked by iberiotoxin and charybdotoxin, Slo1 channel blockers [183]. However, calcium ions stimulated the Slo2-mediated thallium cations uptake [183]. Contrary to mammalian cells, in *C. elegans*, the Slo2 gene product was shown to be Ca^2+^ sensitive [186]. Further studies showed that deletion of the Slo2.2 gene, but not the Slo2.1 gene in mice, resulted in loss of the cardioprotective effect of bithionol- and isoflurane-induced anesthetic preconditioning [187]. On the other hand, the same study showed that ischemic preconditioning-induced cardioprotection was independent of the presence of Slo2 channels. The same was observed for diazoxide-induced cardioprotection [187]. Thallium cations transport stimulated by isoflurane was disturbed in mitochondria isolated from mouse hearts with deletion of both Slo2.1 and Slo2.2 or exclusively Slo2.1. By contrast, deletion of single genes, either Slo2.1 or Slo2.2, did not change Tl^+^ uptake in mitochondria stimulated by bithionol; however, in double knockout, the Tl^+^ uptake induced by this modulator was reduced [187]. However, after deletion of Slick, bithionol still induced cardioprotection. It was proposed that this modulator could stimulate mitochondrial function and cardioprotective effects via alternative targets [187].

Functional data suggesting the important role of Slo2 channels in cardioprotection were recently supported by a study showing mitochondrial localization of these channels in cardiac cells [188]. Mitoplast patch-clamp experiments revealed the channel that had 138 pS conductance. This activity was absent in the Slick deletion mitochondria. The channel was activated by sodium ions and bithionol [188]. It was also found that these channels contribute to the mitochondrial physiology. However, no significant difference was found in mitochondrial ultrastructure, mass or content between the wild-type and Slick knockout mitochondria. On the other hand, it was observed that mitochondria lacking K_Na_1.2 had lower respiratory reserve (defined as the maximal mitochondrial respiration after uncoupler application). This effect was visible when palmitate was used as a respiratory substrate. Therefore, it was concluded that loss of K_Na_1.2 channels results in respiratory reserve deficit specific for fat oxidation by cardiac mitochondria. These disturbances can be observed mainly under conditions of high energy demands [188].

In summary, Slick channels, rather than Slack channels, are another target for cardioprotection. A decrease in intracellular pH during ischemia is a promoting factor for the imbalance of other cations, especially Na^+^ (known as Na^+^ overload) [26]. Together with mitoBK_Ca_ channels, they might play a role in anesthetic-stimulated heart preconditioning. Additionally, it was proposed that activation of mitochondrial Slick channels may be upstream of mitoK_ATP_ channels during ischemic preconditioning.

## 6. Voltage-Regulated Potassium Channels

In addition to the calcium-activated potassium channels, cardiac mitochondria also contain voltage-regulated potassium channels. This was concluded based on several experiments. In adult rat heart lysates and mitoplasts, Western blot analysis revealed the presence of the Kv7.4 pore-forming subunit [8]. This finding was confirmed by analyzing the mRNA transcripts. The pore-forming subunit of Kv7.4 was also detected in the mitochondria of H9c2 rat cardiomyoblast cells. Interestingly, the signal was absent in cytosolic and microsomal fractions, which might suggest that the mitochondrial localization of the channel is exclusive in this cell line. However, in the cytosolic fraction Kv7.1 protein was detected in cardiac myoblasts. Additionally, the presence of Kv7.4 in H9c2 cell mitochondria and acutely isolated rat adult primary cardiomyocytes was confirmed by the use of immunofluorescence and electron microscopy experiments [8]. Functional studies using isolated mitochondria revealed that the application of the Kv7.2–7.5 activators retigabine and flupirtine stimulated the influx of thallium cations into the mitochondrial matrix. This effect was abrogated by the selective Kv7 blocker XE991. This effect was not observed in hepatic mitochondria where the Kv7.4 channel was not detected. Activators of the channel were seen to induce the depolarization of mitochondria in an XE991-dependent manner. Similar effects were observed in the mitochondria of H9c2 cells [8]. Interestingly, activation of the channel by pharmacological modulators prevented calcium influx into the mitochondria. This effect was blocked by the application of a Kv7.4 inhibitor. On the other hand, the channel opener induced the mitochondrial ROS in an XE991-dependent manner. The above data provide evidence for the presence of Kv7.4 channels in the mitochondrial fraction of cardiac cells.

The application of retigabine significantly increased H9c2 cell viability after anoxia/reoxygenation. It was concluded that ROS involvement is not crucial in the cytoprotection induced by this opener. Activation of the channel by retigabine during the pre-ischemic phase was seen to induce cytoprotection in Langendorff-perfused adult rat hearts in the ischemia/reperfusion experiments. The beneficial effects of the channel opener were abolished by the earlier pretreatment of the tissue with XE991. It was also observed that application of the channel opener during the reperfusion phase did not protect the heart tissue [8].

## 7. Summary and Perspective

Why is the simple process of potassium ion influx into cardiac mitochondria carried out by several different mitochondrial potassium channels? As we described above, ATP, calcium and sodium ions, voltage, pH and redox signals regulate mitoK activity in the heart. All these factors are also crucial for the regulation and preservation of the mitochondrial function during heart failure. Therefore, mitochondria have channel proteins that can react to each of these factors with proper timing and directly affect their functions through the same, simple mechanism based on the flow of potassium ions through the inner mitochondrial membrane. Probably, the list of potassium channels present in the cardiac mitochondria could be expanded. A recent report indicates the potential presence of the HCN family channels in the mitochondria of the rat heart [189]. However, these proteins are waiting for more detailed characterization.

Furthermore, a detailed understanding of the regulation and the role of multiple mitochondrial potassium channels in cardiac mitochondria will probably help to describe the role of these proteins in cardioprotection. Different mitochondrial channel proteins in cardiac tissue will probably allow us to interfere with various steps of heart failure. Additionally, it will rationalize the application of specific potassium channel modulators as tools for preventive pharmacological intervention in ischemia/reperfusion injury.
